# A Randomized, Double-Blind, Placebo-Controlled Study to Evaluate the Effectiveness of a Food Supplement Containing Creatine and D-Ribose Combined with a Physical Exercise Program in Increasing Stress Tolerance in Patients with Ischemic Heart Disease

**DOI:** 10.3390/nu11123075

**Published:** 2019-12-17

**Authors:** Giuseppe Derosa, Silvia Pasqualotto, Gabriele Catena, Angela D’Angelo, Antonio Maggi, Pamela Maffioli

**Affiliations:** 1 Department of Internal Medicine and Therapeutics, University of Pavia and Fondazione IRCCS Policlinico San Matteo, 27100 Pavia, Italy; labmedmol@smatteo.pv.it (A.D.); pamelamaffioli@hotmail.it (P.M.); 2 Center for the Study of Endocrine-Metabolic Pathophysiology and Clinical Research, University of Pavia, 27100 Pavia, Italy; 3 Laboratory of Molecular Medicine, University of Pavia, 27100 Pavia, Italy; 4 Section of Cardiology, Department of Medicine, University of Verona, 37017 Verona, Italy; silvia.pasqualotto@libero.it; 5 Cardiologic Unit, ASL of Teramo, 64100 Teramo, Italy; cardio28@gmail.com; 6 Cardiologic Unit, Poliambulanza Foundation, 25020 Brescia, Italy; antonio.maggi@poliambulanza.it

**Keywords:** creatine, cardiovascular disease, D-ribose

## Abstract

The aim of this study is to establish whether a supplement of creatine and ribose combined with a physical exercise program can improve the total work capacity during exercise in a population of patients with known ischemic heart disease. A double-blind, six-month study was designed in which 53 patients were enrolled and randomized to take either a nutraceutical composition containing creatine, D-ribose, vitamin B_1_, and vitamin B_6_ (active treatment) or the placebo. Both the nutraceutical supplement and the placebo were supplied by Giellepi S.p.A. Health Science in Lissone, Italy. After six months of study, the cardiac double product at the peak of the load, the delta double product, and the chronotropic index were higher in the active treatment group than in the placebo group. We can conclude that a supplementation with creatine, D-ribose, vitamin B_1_, and vitamin B_6_, in addition to standard therapy and a physical exercise program, seems to be helpful in improving exercise tolerance compared to the placebo in a population with cardiovascular disease. However, this needs to be further studied, given that there is no clear evidence that the double product can be used as a surrogate measure of exercise tolerance.

## 1. Introduction

Cardiovascular diseases (CVD) are among the most significant causes of death and disabilities worldwide [1]. There is interest in the development of new products, such as dietary supplements and nutraceuticals, for identifying and developing integrative therapies for CVD [2,3]. As suggested by the literature, certain nutritional components (creatine [4], ribose [5,6]) can enhance the energy levels for the heart muscle function and the body’s antioxidant capacity through the reduction of free-radical activity, which is one of the main pathogenic mechanisms of these diseases [7]. Creatine (Cr) is an amino acid that is naturally present in humans. It is mainly accumulated in the skeletal muscle (95%), in free form (40%), and in the form of phosphocreatine (PCr) (60%) [8]. The creatine/creatine kinase (Cr/CK) reaction contributes to produce energy for cardiac muscle cells, and the energetic state of the heart is commonly reported as the PCr/ATP ratio [9]. During myocardial ischemia, tissue hypoxia induces a rapid depletion of PCr and ATP levels [10]. Multiple studies have shown a reduced PCr/ATP ratio in patients with dilated cardiomyopathy [11], even prior to overt cardiac dysfunction, suggesting a close association between cardiac energy status and function [12]. In vivo studies using phosphorous magnetic resonance spectroscopy after myocardial infarction showed that CK flux is reduced in ischemic myocardium, commensurate with the extent of infarct transmurality [13]. Moreover, patients treated with intravenous PCr had reduced incidence of both ventricular fibrillation and ventricular tachycardia post myocardial infarction [14].

Ribose is a 5-carbon monosaccharide and is used in several metabolic pathways that increase available energy for the cell [15]. There are several studies that demonstrate the utility of this sugar in improving cardiac reperfusion after arterial bypass [16], reducing the harm associated with myocardial ischemic events [17], and supporting the energy properties of the heart in congestive heart failure [5]. Moreover, studies have reported a deficiency of high-energy phosphate (ATP) in ischemic heart disease [9,18]. Researchers have demonstrated that a temporal correlation exists between myocardial ATP levels and the diastolic function following myocardial ischemic insult [9]. Supplementation with D-ribose produced a rapid return in ATP levels, as well as an improvement in diastolic dysfunction caused by ischemia [19]. Clinically, Pliml et al. assessed that daily doses of D-ribose in patients with stable severe coronary artery disease can increase their “ischemic threshold”, which is reflected in their ability to exercise longer with fewer symptoms or potential electrocardiographic changes [20]. Most recently, another study investigated the role of D-ribose in congestive heart failure patients with preserved systolic function and diastolic dysfunction. The researchers found an improvement in the tissue Doppler velocity (E′) and an improvement in the ratio of early diastolic filling velocity (E) to early annulus relaxation velocity (E′). Four patients also had an improvement in their maximum predicted VO2 values [21].

Within this context, our research is designed with the principal purpose of establishing whether a supplement of creatine and ribose, in addition to a physical exercise program, can improve the total work capacity during exercise in a population of patients with known ischemic heart disease.

## 2. Material and Methods

### 2.1. Patient Selection

We enrolled 53 patients attending the clinic for Secondary Prevention for Cardiovascular Disease in the Hospital of Verona (Coordinating site) and the Centre of Diabetes and Metabolic Diseases, Department of Internal Medicine and Therapeutics, University of Pavia and Fondazione IRCCS Policlinico in San Matteo, PAVIA, Italy. Among the 53 patients identified, 42 agreed to participate in this research and signed the informed consent form.

The study protocol was approved by the Ethical Committee for Clinical Trials of Verona with the protocol number 23473 on May 11, 2016. TRIAL REGISTRATION: ClinicalTrials.gov NCT03411369.

The trial was conducted in accordance with the 1994 Declaration of Helsinki (Proposed International Guidelines for Biomedical Research Involving Human Subjects, 1982) and its amendments, and the Code of Good Clinical Practice. All patients provided written informed consent to participate in this study after a full explanation of the study.

The first patient was enrolled on July 5, 2016, and that patient’s last visit took place on May 21, 2018. To be included in the study, patients had to have a history of previous acute coronary syndrome and a discharge from the hospital dated at least 30 days before study enrolment. Exclusion criteria were oncological diseases, stable atrial fibrillation, stent in the common core, patients who are not able to perform physical activities, patients with documented sustained ventricular arrhythmias, and pregnant women. All patients were tracked in a lab designed for the secondary prevention of cardiovascular disease. Secondary prevention is defined as a series of coordinated actions designed to eliminate or minimize the impact of cardiovascular disease and related disabilities on an individual’s life through lifestyle changes that include proper nutrition, constant and controlled physical activity, and reduction of risk factors [21].

During the program, the treatment regimen of all patients was optimized in accordance with the European Society of Cardiology guidelines. Standardized pharmacological treatment during the cardiac rehabilitation period included administration of angiotensin converting enzyme inhibition (ACE), beta-blockers and antiplatelet blockers. Furthermore, in order to keep patients free from angina symptoms, calcium channel blockers and/or nitrates were also used in some cases.

### 2.2. Study Design

We randomized patients into two groups in our double-blind, six-month study; one group received active treatment [Creatine, D-Ribose, B_1_ Vitamin, and B_6_ vitamin] and the other received the placebo. Both the nutraceutical supplement and the placebo were supplied by Giellepi S.p.A. Health Science in Lissone, Italy.

We evaluated the following at the baseline and at the end of the study: The maximum load tolerated (expressed in Watts) during cycle ergometer stress testing, clinical exams, body composition in % FM, free fat mass (FFM), % water (with Tanita DC430), blood analysis with lipid profile [TC, LDL-C, HDL-C) and Tg], blood glucose and creatinine. We also performed a cardiologic visit and an ECG at rest.

### 2.3. Stress Test at Cycle Ergometer and Physical Activity

Each participant, following cardiologist authorization, underwent an incremental cycle ergometer test with the workload increased at a ramped rate of 25 W.min-1 until reaching exhaustion. This was done at the baseline and after 6 months. Heart rate, blood pressure, and ECG were continuously recorded.

We gave a pedometer to each patient for monitoring physical activity at home, and we suggested archiving at least 10,000 steps/day. There was also the possibility (not mandatory to be enrolled in the study) to perform supervised physical activity in the lab after obtaining additional consent from the patient. The supervised sessions were performed weekly for 11 weeks and consisted of 30 min of cycling + stretching exercises. The load was set at 65%–70% of the maximum load tolerated for the previously-done exercise test. During the session, the load was increased by 5 watts after 5 min and 15 min. After 25 min, the load was returned to the initial values for the cool down. The patients were questioned using the Borg scale at 4, 10, 20, and 29 min. HR was monitored by a heart rate monitor at 4, 10, and 25 min, and after the cool down. BP was monitored at the beginning of the session, at 25 min and after the cool down. The increase in load from one session to the other was determined by the investigator based on the Borg scale, FC and PA values from the previous session.

### 2.4. Creatine, D-ribose, Vitamin B_1_, and Vitamin B_6_ Supplement

The active dietary supplement comes in the form of water-soluble powder in 4-gram sachets. Each sachet contains 1 gram of creatine, 2.5 grams of D-ribose, 0.33 mg of vitamin B_1_, and 0.42 mg of vitamin B_6_. The treatment consists of taking 2 sachets/day for the first two weeks, and then continuing with 1 sachet/day for the next month. The same administration was given to the placebo group with inert product sachets containing starch powder. To assess the adherence to therapy at the end of the study, all patients were required to turn in the empty boxes and sachets of active treatment and placebo used.

### 2.5. Statistics Analyses

The Shapiro–Wilk test was performed for testing the normality of data. The variables were compared using a Student’s *t*-test for unpaired data and analysis of variance for repeated measures (ANOVA). The level of significance was *p* < 0.05. Statistical analysis of data was performed by the SPSS statistical software package for Windows (version 21.0; Chicago, IL, USA). The Shapiro–Wilk test was performed for testing the normality of data.

## 3. Results

### 3.1. Study Sample

Over the study period, 53 patients were screened for eligibility. However, before entering the randomization process, 11 patients withdrew from the study, while three patients (one from the active treatment group and two from the placebo group) dropped out after 2, 8, and 15 weeks of treatment for personal reasons. Ultimately, 42 patients were enrolled and randomly assigned to receive either active treatment (*n* = 21) or the placebo (*n* = 21). At the end of the protocol, each patient underwent clinical evaluation and a cycle ergometer stress test.

Cardiovascular disease etiology and risk factors in the total population, placebo, and active treatment groups are described in Table 1. The treatment period was similar between the two groups: 171 ± 14 days for the placebo group and 172 ± 10 days for the active treatment group. Out of the total number of patients, 54.8% decided to carry out supervised physical activity in the lab. With regard to home-based physical activity monitored by a step counter, increased exercise was reported in both groups, without significant differences between them (at the end of the study, the step counter average per day was 10238 ± 5188 steps in the placebo group and 10,327 ± 2985 steps in the active treatment group (Tables 5 and 7).

The adherence to trial treatment, evaluated by counting the packets of active treatment or placebo that patients used, was 87% in the placebo group and 90% in the active treatment group. No adverse reactions were reported.

### 3.2. Clinical Characteristics Parameters During the Study

Regarding clinical characteristics at the baseline (Table 2), the two groups were similar with the exception of moderately higher values of body weight, Body Mass Index (BMI) and % Fat Mass (FM) in patients belonging to the active treatment group (*p* = 0.04 for all), and a higher % of water in patients from the placebo group (*p* = 0.03).

Table 3 shows the results obtained by comparing the basal and final results within each group. We recorded a significant decrease in total cholesterol (TC) and low density lipoprotein cholesterol (LDL-C) in both groups. A significant increase of high density lipoprotein cholesterol (HDL-C) and a significant decrease of triglycerides (Tg) (*p* = 0.01 and *p* = 0.04 respectively) were observed in the active treatment group only. A reduction of alanine aminotransferase (ALT) (*p* = 0.01) was observed in the placebo group only.

Comparing the results obtained in the two groups after six months of treatment (Table 4), the differences in body composition initially noted were no longer significant, while we noticed a slightly higher value of creatinine in the active treatment group compared to the placebo group (*p* = 0.04).

### 3.3. Stress Test Parameters During Incremental Cycle Ergometer Stress Testing 

Stress test parameters during incremental cycle ergometer stress testing at basal evaluation are listed in Table 5. Furthermore, in this case, the stress test parameters were similar between the groups, with the exception of a lower baseline systolic blood pressure in the active treatment group (*p* = 0.03) and a higher metabolic equivalent value (METs) in the placebo group (*p* = 0.05). The duration of the cycle ergometer stress test at baseline was similar between the two treatments: 7.88 ± 1.85 min for the placebo group and 7.12 ± 2.31 min for the active treatment group. Moreover, the step count (average per day) did not differ between groups at the baseline; there were 7685 ± 2854 steps in the placebo group and 8393 ± 3720 steps in the active treatment group.

Considering the results between the baseline and the ones obtained at the end of the study inside each group (Table 6), the duration of the cycle ergometer stress test was longer in both arms [from 7.99 ± 1.88 to 8.96 ± 1.55 min (*p* = 0.05) in the placebo group, and from 7.15 ± 2.31 to 8.94 ± 2.83 min (*p* = 0.04) in the active treatment group]. On the other hand, the maximum load tolerated increased significantly from 98 ± 33 to 126 ± 37 watts (*p* = 0.02) in the active treatment group only.

As concern parameters indirectly associated with myocardial VO2 and cardiac mortality, the double heart product at the peak of the load increased in both groups [from 17,425 ± 3882 to 20,611 ± 3997 (*p* = 0.02) in the placebo group, and from 18,672 ± 3631 to 23,375 ± 4171 (*p* = 0.003) in the active treatment group]. The same trend was observed for the delta double product, which increased from 9.78 ± 3.33 to 13.35 ± 3.25 (*p* = 0.003) in the placebo group, and from 11.41 ± 2.91 to 15.75 ± 4.09 (*p* = 0.001) in the active treatment group. The chronotropic index, which represents the fraction of chronotropic reserve during exercise, was significantly increased in both groups, from 0.52 ± 0.17 to 0.64 ± 0.18 (*p* = 0.01) in the placebo group, and from 0.54 ± 0.13 to 0.74 ± 0.12 (*p* = 0.00001) in the active treatment group.

Comparing the results obtained during the first evaluation and those obtained after six months of treatments between the two groups (Table 7), the double product at the peak of the load [23,374 ± 4168 vs. 20,611 ± 3996 (*p* = 0.04)], the delta double product [15.76 ± 4.11 vs. 13.24 ± 3.22 (*p* = 0.03)] and the chronotropic index [0.74 ± 0.11 vs. 0.67 ± 0.21 (*p* = 0.046)] were higher in the active treatment group than in the placebo group. These parameters, as mentioned above, are very important as they are indirectly associated with myocardial VO2 and cardiac mortality.

## 4. Discussion

The clinical benefits of exercise are the result of a complex web of interrelated physiologic mechanisms, each having a potentially unique exercise dose–response relationship. Some of these physiologic responses and adaptations to exercise include the following: improved weight control and body composition, improved lipid and lipoprotein profiles, improved blood glucose homeostasis and insulin sensitivity, improved fibrinolysis and thrombolysis capacity, improved coronary blood flow and arrhythmic threshold, improved endothelial function and blood pressure regulation, and lower levels of circulating inflammatory and thrombogenic factors [21]. Longitudinal studies involving individuals with CVD show that aerobic exercise training in combination with diet and other risk-factor interventions can prevent the progression, and perhaps reduce the severity, of atherosclerosis [22]. In our study, the lipid profile improvements observed within each group may be due to changes in the lifestyle of our patients, which was strictly controlled from a nutritional point of view and supported by carrying out regular physical activity (at least 10,000 steps/day). Our outcomes are in line with other studies and confirm that a secondary prevention program positively affects the reduction of cardiovascular risk factors. In their GOSPEL study, Giannuzzi et al. also demonstrated that an integrated, multifactorial, reinforced, and individually tailored secondary prevention program is effective in reducing major CVD events. Indeed, a greater proportion of patients in their intervention group achieved the expected targets for physical activity, healthy diet, stress management, weight reduction, triglyceride and HDL cholesterol levels, and blood pressure [23]. Chlow et al. also found that adherence to recommendations regarding smoking, diet, and exercise are associated with a substantially lower rate of short-term major cardiovascular outcomes and all-cause mortality. In particular, those who adhered to diet and exercise recommendations had a 50% lower risk for all major events in six months compared with those who did not [24].

Regarding stress test parameters, it is well known that in patients who have undergone a myocardial infarction, an adequate cardiac rehabilitation program that includes physical exercise may contribute to the reduction of the ischemic area, reduced risk of recurrence for further coronary events and reduced mortality. On the other hand, stress tolerance is increased, and the psycho-social aspect of job placement and everyday life are improved [25,26,27]. The double product is related to the myocardial oxygen consumption [28,29] and its use was justified physiologically during exercise testing in patients with ischemic heart disease. Despite the lack of clarity of the double product beyond its meaning in exercise testing, comparative clinical trials have been done to determine the superiority of one drug over another in reducing the double product, both during the morning surge in blood pressure [30,31] and over a 24-hour period [32]. For example, White et al. [32] conducted a randomized clinical trial for eight weeks to compare the efficacy and tolerability of a calcium-channel blocker (amlodipine besylate) and a beta-blocker (metoprolol succinate) in reducing the double product over 24-hours and in the early morning (upon awakening) in 35 hypertensive patients. The authors concluded that the beta-blocker provided a more effective control of the double product. However, the significant reduction in the 24-hour double product and the morning double product effected by the beta-blocker as compared with the calcium-channel blocker was due to the result of a decrease in the HR. The results obtained in our study confirm that the active treatment could increase the tolerance to physical effort. In particular, we demonstrated an improvement of double product with the active treatment supplementation; this is the first time that a food supplement has demonstrated this with respect to the other drugs mentioned above.

Regarding the mechanism by which the nutraceutical can act, it is well known that the creatine/creatine kinase (Cr/CK) reaction is the major source of energy in cardiac muscle cells, and the energetic state of the heart is commonly reported as the PCr/ATP ratio. Multiple studies have shown a reduced PCr/ATP ratio in patients with dilated cardiomyopathy [33], cardiac dysfunction in hypertension [34], obesity [12], and type 2 diabetes [35], suggesting a close association between cardiac energetic status and function.

In animal models, PCr treatment reduced necrotic tissue injury and improved contractile function during coronary artery ligation [12] and ischemia-reperfusion injury [36], preventing ventricular dysfunction during transient coronary occlusion [37,38]. Cornelissen et al. investigated the effect of a 5 g oral creatine supplementation in conjunction with an exercise program in patients with CVD. This study showed that oral creatine supplementation in combination with exercise training did not exert any additional effect on the improvement in physical performance, health-related quality of life and lipid profile [39]. Regarding supplementation with D-ribose during and following an ischemic insult, this induced a rapid return in ATP levels, as well as an improvement in diastolic dysfunction, due to the ischemia. Numerous studies have further reported on the benefit of D-ribose in replenishing deficient ATP levels following a cellular insult, including myocardial ischemia. Ingwall and Weiss reported that the failing heart is energy starved, and therefore, efforts to increase myocardial energy levels in an energy-compromised myocardium may show promise [9]. Various preclinical animal studies have consistently reported that a deficiency in myocardial ATP levels follows ischemia. This depletion in high-energy phosphates following ischemia has been investigated in isolated rat hearts and in a chronic canine model [40,41]. Ward and colleagues reported that there is an approximate 50% reduction in myocardial ATP levels following a moderate 20-minute global ischemic insult, for which an extended period of time is required for total recovery [9]. Sawada et al. investigated the ability of D-ribose with a low dose of dobutamine to improve the contractile response of viable myocardium, and they also assessed the efficacy of D-ribose in reducing stress-induced ischemia. The study shows that D-ribose improved contractile responses to dobutamine in viable myocardium with resting dysfunction, but had no significant effect in reducing the frequency of stress-induced wall motion abnormalities [42].

Of course, our study has some limitations, such as the short duration of the study or the fact that we did not observe if the effects of the nutraceutical agent were reversible after the interruption of treatment. Moreover, additional physical activity was done during the study by some patients, and this might create bias even if the step count was similar between the two groups at the end of the study. Finally, previous studies [38] have shown that the administration of exogenous creatine was unable to increase creatine content in the myocardium. Given that we did not measure myocardial creatine changes in enrolled patients, we cannot exclude that the possible creatine benefits may have been due to improvement in skeletal muscular metabolism, perhaps via the physical exercise program that was carried out jointly with supplementation.

## 5. Conclusions

Supplementation with creatine, D-ribose, vitamin B_1_, and vitamin B_6_, in addition to standard therapy and a physical exercise program, seems to be helpful and to improve exercise tolerance compared to the placebo in a population with cardiovascular disease tracked in a secondary prevention program. However, future studies will be required to confirm these preliminary data and to better understand the mechanism underlying this effect, given that there is no clear evidence that the double product can be used as a surrogate measure of exercise tolerance.

## Figures and Tables

**Table 1:** Cardiovascular disease etiology and risk factors in total population, placebo and active treatment groups.

	Total Population (*n* = 42)	Placebo Group (*n* = 21)	Active Treatment Group (*n* = 21)
CVD etiology (n, %)			
STEMI	24 (57.1)	14 (66.7)	10 (47.6)
Non-STEMI	18 (42.9)	7 (33.3)	11 (52.4)
Risk factors (n, %)			
Smoker	18 (42.9)	10 (47.6)	8 (38.1)
Former smoker (>15 years)	11 (26.2)	7 (33.3)	4 (19.0)
HT	21 (50)	13 (61.9)	10 (47.6)
DM	5 (11.9)	2 (9.5)	3 (14.3)
IFG	7 (16.7)	5 (23.8)	2 (9.5)
Glucose intolerance	2 (4.8)	3 (14.3)	/
Dyslipidemia	19 (45.2)	10 (47.6)	11 (47.6)
Hypercholesterolemia	3 (7.1)	2 (9.5)	/
Familiarity	16 (38.1)	8 (38.1)	7 (33.3)

Abbreviations: CVD: Cardiovascular disease; STEMI: ST-Elevation myocardial infarction; HT: Hypertension; DM: Diabetes mellitus; IFG: Impaired fasting glucose.

**Table 2:** Comparison of clinical parameters at basal evaluation between the placebo and active treatment groups.

Parameters	Placebo (*n* = 21)	Active Treatment (*n* = 21)	*p*-Value
Age (years)	55.76 ± 7.76	59.46 ± 9.72	0.14
Weight (Kg)	77.54 ± 11.08	83.18 ± 14.86	0.04
Height (m)	1.72 ± 0.08	1.69 ±0.08	0.18
BMI (Kg/m^2^)	26.26 ± 3.41	29.01 ± 4.28	0.04
% FM	25.96 ± 4.51	31.72 ± 9.15	0.04
% FFM	69.46 ± 19.15	64.89 ± 18.99	0.26
% Water	53.89 ± 3.75	51.08 ± 3.14	0.03
TC (mg/dL)	161.28 ± 52.04	179.05 ± 55.90	0.21
LDL-C (mg/dL)	88.79 ± 45.33	102.09 ± 34.54	0.18
HDL-C (mg/dL)	43.28 ± 12.45	39.43 ± 11.48	0.19
Tg (mg/dL)	146.55 ± 80.34	183.29 ± 169.11	0.25
Creatinine (mg/dL)	0.99 ± 0.16	1.17 ± 0.66	0.13
ALT (U/L)	58 ± 37	78 ± 22	0.28
Blood glucose (mg/dL)	108.76 ± 18.25	121.52 ± 35.64	0.11

Abbreviations: BMI: Body Mass Index; FM: Fat Mass; FFM: Free Fat Mass; LDL cholesterol: Low-density lipoprotein cholesterol; HDL cholesterol: High-density lipoprotein cholesterol; ALT: Alanine transaminase.

**Table 3:** Difference of clinical characteristics between the basal and final evaluation for each group.

Parameters	Baseline Evaluation	6 Months Evaluation	*p*-Value
Weight (Kg)			
Placebo	77.54 ± 11.08	79.15 ± 10.08	0.38
Active treatment	83.18 ± 14.86	80.59 ± 14.06	0.31
Height (m)			
Placebo	1.72 ± 0.08	1.71 ± 0.08	0.41
Active treatment	1.69 ± 0.08	1.68 ± 0.08	0.4
BMI (Kg/m^2^)			
Placebo	26.26 ± 3.41	27.01 ± 3.31	0.32
Active treatment	29.01 ± 4.28	28.61 ± 4.15	0.39
% FM			
Placebo	25.96 ± 4.51	27.67 ± 3.55	0.3
Active treatment	31.72 ± 9.15	26.37 ± 4.59	0.04
% FFM			
Placebo	9.46 ± 19.15	71.76 ± 4.91	0.35
Active treatment	64.89 ± 18.99	69.75 ± 5.71	0.19
% Water			
Placebo	53.89 ± 3.75	52.31 ± 4.14	0.16
Active treatment	51.08 ± 3.14	50.82 ± 4.41	0.48
TC (mg/dL)			
Placebo	161.28 ± 52.04	123.65 ± 26.04	0.01
Active treatment	179.05 ± 55.90	122.03 ± 20.41	0.001
LDL-C (mg/dL)			
Placebo	88.79 ± 45.33	52.88 ± 18.69	0.005
Active treatment	102.09 ± 34.54	56.63 ± 18.03	0.0001
HDL-C (mg/dL)			
Placebo	43.28 ± 12.45	48.04 ± 19.35	0.22
Active treatment	39.43 ± 11.48	49.94 ± 10.95	0.01
Triglycerides (mg/dL)			
Placebo	146.55 ± 80.34	113.67 ± 79.67	0.14
Active treatment	183.29 ± 169.11	92.26 ± 43.41	0.04
Creatinine (mg/dL)			
Placebo	0.99 ± 0.16	0.98 ± 0.17	0.45
Active treatment	1.17 ± 0.66	1.14 ± 0.26	0.44
ALT (U/L)			
Placebo	58 ± 37	34 ± 20	0.01
Active treatment	78 ± 22	43 ± 21	0.16
Blood glucose (mg/dL)			
Placebo	108.76 ± 18.25	105.25 ± 11.31	0.29
Active treatment	121.52 ± 35.64	111.18 ± 17.91	0.18

Abbreviations: BMI: Body Mass Index; FM: Fat mass; FFM: Free fat mass; LDL cholesterol: Low-density lipoprotein cholesterol; HDL cholesterol: High-density lipoprotein cholesterol; ALT: Transaminases.

**Table 4:** Comparison of clinical characteristics after six months of treatment.

Parameters	Placebo (*n* = 19)	Active Treatment (*n* = 20)	*p*-Value
Weight (Kg)	79.15 ± 10.08	80.59 ± 14.06	0.35
Height (m)	1.71 ± 0.08	1.68 ± 0.08	0.19
BMI (Kg/m^2^)	27.01 ± 3.31	28.61 ± 4.15	0.13
% FM	27.67 ± 3.55	26.37 ± 4.59	0.29
% FFM	71.76 ± 4.91	69.75 ± 5.71	0.23
% Water	52.31 ± 4.14	50.82 ± 4.41	0.19
TC (mg/dL)	123.65 ± 26.04	122.03 ± 20.41	0.47
LDL-C (mg/dL)	52.88 ± 18.69	56.63 ± 18.03	0.35
HDL-C (mg/dL)	48.04 ± 19.35	49.94 ± 10.95	0.38
Tg (mg/dL)	113.67 ± 79.67	92.26 ± 43.41	0.22
Creatinine (mg/dL)	0.98 ± 0.17	1.14 ± 0.26	0.04
ALT (U/L)	34 ± 20	43 ± 21	0.06
Blood glucose (mg/dL)	105.25 ± 11.31	111.18 ± 17.91	0.17

Abbreviations: BMI: Body Mass Index; FM: Fat mass; FFM: Free fat mass; LDL cholesterol: Low-density lipoprotein cholesterol; HDL cholesterol: High-density lipoprotein cholesterol; ALT: Alanine transaminase.

**Table 5:** Comparison of clinical parameters during incremental cycle ergometer stress testing at basal evaluation between the placebo and active treatment groups.

Parameters	Placebo (*n* = 21)	Active Treatment (*n* = 21)	*p*-Value
Exercise duration (min)	7.99 ± 1.88	7.15 ± 2.31	0.13
Maximum load tolerated (Watt)	115 ± 28	98 ± 33	0.08
Theoretical HR max (bpm)	162 ± 9	163 ± 12	0.14
HR baseline (bpm)	64 ± 9	66 ± 17	0.37
SBP baseline (mmHg)	121 ± 15	110 ± 12	0.03
Double product baseline	7664 ± 1442	7291 ± 2075	0.26
HR at peak (bpm)	112 ± 13	115 ± 14	0.48
SBP at peak (mmHg)	153 ± 22	166 ± 23	0.12
Double product at peak	17,425 ± 3882	18,672 ± 3631	0.16
DBP at peak (mmHg)	83 ± 11	82 ± 10	0.20
Δ Double product	9.78 ± 3.33	11.41 ± 2.91	0.09
METs	6.61 ± 2.25	5.45 ± 1.31	0.05
METs predicted	9.67 ± 1.21	9.12 ± 1.49	0.12
% METs	69 ± 24	63 ± 17	0.12
Chronotropic index	0.52 ± 0.17	0.54 ± 0.13	0.41
SBP 3′ recovery	132 ± 23	134 ± 14	0.41
SBP 3′ recovery/SBP at peak	0.87 ± 0.11	0.79 ± 0.06	0.09
HR 1′ recovery	95 ± 13	92 ± 15	0.40
D HR at peak/HR 1′ recovery	1.23 ± 0.12	1.26 ± 0.18	0.30
Step count (average per day)	7685 ± 2854	8393 ± 3720	0.26

Abbreviations: HR: Heart rate; SBP: Systolic blood pressure; DBP: Diastolic blood pressure; MET: Metabolic equivalent.

**Table 6:** Difference between stress test parameters for basal and final evaluations for each group.

Parameters	Baseline Evaluation	6 Months Evaluation	*p*-Value
Exercise duration (min)			
Placebo	7.99 ± 1.88	8.96 ± 1.55	0.05
Active treatment	7.15 ± 2.31	8.94 ± 2.83	0.04
Maximum load tolerated (Watt)			
Placebo	115 ± 28	128 ± 23	0.08
Active treatment	98 ± 33	126 ± 37	0.02
HR baseline (bpm)			
Placebo	64 ± 9	64 ± 7	0.37
Active treatment	66 ± 17	66 ± 11	0.48
SBP baseline (mmHg)			
Placebo	121 ± 15	116 ± 17	0.36
Active treatment	110 ± 12	115 ± 13	0.11
Double product baseline			
Placebo	7664 ± 1442	7502 ± 1711	0.38
Active treatment	7291 ± 2075	7639 ± 1610	0.3
HR at peak (bpm)			
Placebo	112 ± 13	124 ± 17	0.03
Active treatment	115 ± 14	132 ± 13	0.0004
SBP at peak (mmHg)			
Placebo	153 ± 22	163 ± 20	0.08
Active treatment	166 ± 23	173 ± 27	0.08
Double product at peak			
Placebo	17,425 ± 3882	20,611 ± 3997	0.02
Active treatment	18,672 ± 3631	23,375 ± 4171	0.003
DBP at peak (mmHg)			
Placebo	83 ± 11	91 ± 9	0.05
Active treatment	82 ± 10	92 ± 13	0.01
Δ Double product			
Placebo	9.78 ± 3.33	13.35 ± 3.25	0.003
Active treatment	11.41 ± 2.91	15.75 ± 4.09	0.001
METs			
Placebo	6.61 ± 2.25	6.50 ± 1.28	0.48
Active treatment	5.45 ± 1.31	6.17 ± 1.35	0.09
METs predicted			
Placebo	9.67 ± 1.21	9.68 ± 1.24	0.52
Active treatment	9.12 ± 1.49	9.01 ± 1.44	0.42
% METs			
Placebo	69 ± 24	68 ± 16	0.45
Active treatment	63 ± 17	71 ± 20	0.08
Chronotropic index			
Placebo	0.52 ± 0.17	0.64 ± 0.18	0.01
Active treatment	0.54 ± 0.13	0.74 ± 0.12	0.00001

Abbreviations: HR: Heart rate; SBP: Systolic blood pressure; DBP: Diastolic blood pressure; MET: Metabolic equivalent.

**Table 7:** Comparison of stress test parameters after six months of treatment.

Parameters	Placebo (*n* = 19)	Active Treatment (*n* = 20)	*p*-Value
Exercise duration (min)	8.96 ± 1.55	8.94 ± 2.83	0.48
Maximum load tolerated (watt)	128 ± 23	126 ± 37	0.43
HR baseline (bpm)	64 ± 7	66 ± 11	0.24
SBP baseline (mmHg)	116 ± 17	115 ± 13	0.41
Double product baseline	7502 ± 1711	7639 ± 1610	0.40
HR at peak (bpm)	124 ± 17	132 ± 13	0.09
SBP at peak (mmHg)	163 ± 20	173 ± 27	0.08
Double product at peak	20,611 ± 3997	23,375 ± 4171	0.04
DBP at peak (mmHg)	91 ± 9	92 ±13	0.30
Δ Double product	13.35 ± 3.25	15.75 ± 4.09	0.03
METs	6.50 ± 1.28	6.17 ± 1.35	0.27
METs predicted	9.68 ± 1.24	9.01 ± 1.44	0.13
% METs	68 ± 16	71 ± 20	0.45
Chronotropic index	0.64 ± 0.18	0.74 ± 0.12	0.046
SBP 3′ recovery	134 ± 23	138 ± 16	0.22
SBP 3′ recovery/SBP at peak	0.89 ± 0.12	0.83 ± 0.15	0.36
HR 1′ recovery	103 ± 17	111 ± 15	0.11
Δ HR peak /HR 1′ recovery	1.26 ± 0.15	1.25 ± 0.18	0.44
Step count (average per day)	10,238 ± 5188	10,327 ± 2985	0.49

Abbreviations: HR: Heart rate; SBP: Systolic blood pressure; DBP: Diastolic blood pressure; MET: Metabolic equivalent.
